# S25. COGNITIVE REMEDIATION THERAPY AND ITS EFFECTS ON BDNF SERUM LEVELS

**DOI:** 10.1093/schbul/sby018.812

**Published:** 2018-04-01

**Authors:** Rafael Penades, Irene López-Vílchez, Rosa Catalán, Barbara Arias, Clemente García-Rizo, Guillem Masana, Miquel Bernardo

**Affiliations:** 1 Hospital Clinic Barcelona, Universitat Barcelona, IDIBAPS, CIBERSAM; 2 Hospital Clínic Barcelona, Universitat Barcelona; 3 Universitat Barcelona; 4 Hospital Clínic Barcelona

## Abstract

**Background:**

Brain-derived neurotrophic factor (BDNF) has been proposed as a biomarker of schizophrenia and, more specifically, as a biomarker of cognitive recovery. Unfortunately, it has only been tested once with cognitive remediation treatment (CRT).

**Methods:**

A randomized and controlled trial (NCT02341131) with 70 schizophrenia outpatients and 15 healthy volunteers was conducted. The participants with schizophrenia were randomly assigned to either CRT or the control group. All the participants were assessed in terms of cognition, quality of life, and their serum BDNF levels at both baseline and after the intervention. Additionally, comparisons of the effects of the different genotypes of the Val66Met polymorphism at the BDNF gene on the outcome variables were also performed.

**Results:**

The patients in the CRT group presented with improvements in cognition. However, no significant changes were detected in the serum levels of BDNF. Interestingly, we found a significant positive interaction effect between the serum BDNF levels and the different BDNF genotypes. The Val/Val group showed significantly higher serum levels after the CRT treatment.

**Discussion:**

The replication of the previous finding of increased serum BDNF levels after cognitive remediation in clinically stable individuals with schizophrenia was not achieved. However, our data indicated that genetic variability may be mediating serum BDNF activity in the context of CRT. All in all, the current consideration of BDNF as a biomarker of cognitive recovery in schizophrenia is promising but still premature.

